# LeafDet: A lightweight and interpretable deep learning framework for tomato leaf disease detection

**DOI:** 10.1371/journal.pone.0349501

**Published:** 2026-05-22

**Authors:** Vaskor Mostafa, Md. Shafak Shahriar Sozol, M. Rubaiyat Hossain Mondal

**Affiliations:** Institute of Information and Communication Technology, Bangladesh University of Engineering and Technology (BUET), Dhaka, Bangladesh; Sultan Qaboos University, OMAN

## Abstract

Ensuring global food security depends on timely and reliable plant disease identification. Traditional disease detection methods often prove inefficient because of the lack of necessary precision. Furthermore, public datasets typically suffer from the class imbalance issue, which can obstruct reliable model testing and lead to biased performance evaluations. This paper introduces LeafDet, an object detection model based on the YOLOv8 architecture, specifically designed for the effective detection of tomato leaf diseases. Moreover, a revised, balanced dataset, named PlantTom, is developed by combining images from various public sources to reduce the existing dataset limitations. PlantTom has 7836 images with 8 distinct classes, each representing a tomato leaf disease. The proposed LeafDet model includes CBM, C2f, SPPF, and ECA attention modules in the backbone section; BiFPN, GSConv, VoVGSCSP, and Shuffle Attention in the neck section. Efficient attention methods like ECA and Shuffle Attention are used to improve both accuracy and speed. LeafDet model achieves 91.6% mAP@0.5 on the PlantTom dataset, which is a 2.2% improvement over the original YOLOv8n with 2.69M parameters and an inference time of 2.4ms. The proposed model also outperforms several other state-of-the-art object detection models, including the latest YOLOv11n and YOLOv12n. Ablation studies show that each part of the model helps to improve its performance, and the PIoUv2 loss function is found to be the optimal choice for this use. The model predictions are then validated using Eigen-CAM, which provides a visualization of the decision-making process. These results demonstrate that LeafDet provides a deployable and interpretable framework for plant disease detection in smart agriculture.

## 1. Introduction

The global population is growing rapidly as well as the food influx is also increasing. To meet this demand and ensure sufficient food for all, it is necessary to produce healthy crops. However, plants are frequently affected by various diseases. This significantly reduces crop production, and farmers face huge economic losses every year. According to a report by the Food and Agriculture Organization (FAO) of the United Nations, every year up to 40% of crops are lost due to plant pests and diseases, and it costs the global economy more than USD220 billion per year [1]. This is a common and devastating problem worldwide. In the past, identifying these diseases depended mostly on manual checking by the experts. This method is slow, unreliable, sometimes inaccurate, and usually finds the disease too late, after it has already spread. If a disease is not identified quickly and reliably, it can spread rapidly. Tomatoes are a globally important crop, so protecting them from diseases has a huge impact on food security and the livelihoods of countless farmers.

To overcome the limitations of the traditional approach, artificial intelligence (AI), especially deep learning (DL) models, can play an important role. When these models are trained on images of healthy and diseased plants, they can learn to recognize disease patterns. This ability makes them effective tools for automated disease detection. A report from FAO describes AI as “game-changing solutions for farmers” [2]. However, even with the rise of DL, there are still challenges to overcome. One major issue is that the datasets used to train these models often contain a class imbalance issue. For instance, an image dataset may contain thousands of healthy tomato leaf images, but only a few images of a rare tomato disease. When a DL model is trained on such an imbalanced dataset, it tends to become very good at recognizing healthy images but struggles with rare ones. This can lead to biased results and make it difficult to evaluate how well a model performs at detecting all classes in real-world situations. Another major problem is that most public datasets are class-imbalanced, which means some disease types have many more images than others. As a result, the model’s accuracy can be biased and unreliable. Currently, object detection models like YOLO (You Only Look Once) are mostly used to quickly and reliably find symptoms of plant diseases. Many improved versions of YOLO, like E-TomatoDet [3], YOLOv8-ACCW [4], and TDGA [5], have shown promising performance in detecting leaf diseases. However, most of these models either infer slowly in real-time settings or do not perform well under different lighting and background conditions.

The objectives of this study are as follows:

i. Introduction of a hybrid dataset, PlantTom, for addressing class imbalance and improving model generalization.
ii. Development of a custom deep learning model, LeafDet, for rapid and reliable detection of tomato leaf diseases.
iii. Comparative evaluation against recent state-of-the-art models, along with an ablation study to validate the contribution of integrated modules, i.e., CBM [6], C2f [7], SPPF [7], ECA Attention [8], BiFPN [9], VoVGSCSP [10], GSConv [10], and Shuffle Attention [11].
iv. Integration of an XAI-based Eigen-CAM [12] approach for visualizing model focus, thereby enhancing interpretability and trustworthiness.

This paper is organized as follows: Section 2 discusses the related works on plant leaf disease detection, Section 3 presents the materials and methods, including dataset development, performance metrics, and DL architecture. Section 4 summarizes the experimental results of the proposed model and other findings. Finally, Section 5 concludes the research.

## 2. Related works

This section reviews recent progress in DL-based plant disease detection, focusing on datasets, methodologies, model improvements, and comprehensive surveys.

Recent studies have shown improved YOLO models for plant disease detection. Researchers have enhanced different YOLO versions (v5, v6, v7, v8, v9, v11) to better spot disease in various plants like apples, tomatoes, grapes, and strawberries. These updated models often add special features like attention mechanisms or enhanced convolution modules. They achieve good accuracy (mAP@0.5 > 90%) and usually outperform the standard YOLO variants. These improvements use diverse datasets, helping to create more reliable and efficient tools for farmers to find plant diseases early.

Recent research has widely considered variations of the YOLO architecture for plant disease detection. Tao et al. [13] proposed CEFW-YOLO, which is an improvement of the YOLOv11 model, and achieved 87.3% mAP@0.5, which is a 7.6% improvement over the baseline. Sun et al. [3] proposed a new model called E-TomatoDet, an improvement of the YOLOv8 model, achieving 97.2% mAP@0.5, which is a 4.7% improvement over the baseline. Abulizi et al. [14] introduced the DM-YOLO model, an enhanced version of YOLOv9, achieving 86.4% mAP@0.5, which is a 1.4% improvement over the baseline on the Tomato leaf diseases detection dataset available on the Roboflow platform (Bryan 2023). Miao et al. [15] introduced SerpensGate-YOLOv8 with Dynamic Snake Convolution and Super Token Attention, achieving a 3.3% improvement in mAP@0.5 over standard YOLOv8. Similarly, Ji *et al.* [16] presented a lightweight tomato leaf disease detection model based on adaptive kernel convolution and feature fusion to enhance YOLOv8 by using AKConv and SlimNeck modules and achieving a 94.9% mAP@0.5, which is a 1.9% improvement over the baseline. In this research, the dataset used is the subset of the Plant Village dataset [17] that contains 10,041 images after augmentation with 9 tomato disease classes. Chen et al. [4] introduced an attention-based improved YOLOv8 model called YOLOv8-ACCW, achieving a 92.8% mAP@0.5, which is a 3.1% improvement over the baseline. Li et al. [18] introduced the YOLO-Leaf model, which is an improvement of the YOLOv8 model, and achieves 93.88% mAP@0.5 on Plant Pathology 2020-FGVC7 (FGVC7) [19] and 95.69% mAP@0.5 on Plant Pathology 2021-FGVC8 (FGVC8) [20] datasets. Wang et al. [21] proposed a YOLOv6-based model integrated with CBAM and BiRepGFPN for multi-scale feature fusion, showing a 2.7% improvement over YOLOv6. In another study, Umar et al. [22] improved YOLOv7 by incorporating SimAM and DAiAM modules, along with SIFT and CNN classification. It achieved 98.8% mAP@0.5, which is a 1.2% improvement in mAP@0.5 over standard YOLOv7. Similarly, Wang et al. [5] developed TDGA for tomato leaf disease detection, an improved YOLOv5 variant using global attention and switchable atrous convolution (SAConv) to address the multi-scale target and achieve 91.4% mAP@0.5, which is a 2.93% improvement over standard YOLOv5. Zhong et al. [23] proposed an improvement of the YOLOv8 model by changing the default complete IoU (CIoU) to powerful IoU version 2 (PIoUv2) and achieving an 85.6% mAP@0.5 across nine tomato diseases, which is a 1.3% improvement over standard YOLOv8. Meng et al. [24] proposed the YOLOV5-CBAM-C3TR model, which achieved 73.4% mAP@0.5 on the publicly available apple leaf pathology image dataset [25], which is an increase of 8.25% compared to the original YOLOv5 model. Wang and Liu [26] proposed a model that achieved 92.3% mAP@0.5 on a self-acquired dataset from a tomato cultivation facility. Abid et al. [27] presented a hybrid dataset of five major crops with a total of 19 classes, achieving 98.3% mAP@0.5 on the YOLOv8 model. Luo et al. [28] introduced Lightweight Self-Attention YOLOv8, achieving a 92.6% mAP@0.5, which is a 1.8% improvement over standard YOLOv8. Yang et al. [29] proposed a lightweight YOLOv8s model using depthwise separable convolutions, a dual-path attention gate (DPAG), and a feature enhancement module (FEM). It achieved 93.4% mAP@0.5 on a self-collected tomato leaf disease dataset, which is a 1.5% improvement in mAP@0.5 over standard YOLOv8 while reducing model size and enhancing real-time performance. Liu et al. [30] improved the YOLOv5 model by adding a Hybrid attention mechanism (HAM) and achieved 94.6% mAP@0.5. Zhang et al. [31] proposed a novel DL model for tomato leaf disease by introducing an Asymptotic Non-Local Means algorithm (ANLM) and Multi-channel Automatic Orientation Recurrent Attention Network (M−AORANet). In this research, a 96.47% mAP@0.5 was achieved on a hybrid tomato leaf disease dataset. Chen et al. [32] proposed an improved lightweight YOLOv5 model and achieved 94.7% mAP@0.5 on a hybrid strawberry disease detection dataset. Zhu et al. [33] proposed an improvement of the YOLOv5 model called EADD-YOLO and achieved a 95.5% mAP@0.5.

In Table 1, an overview of recent works is presented, highlighting the used models and datasets, number of images and classes, along with their reported performance metrics (mAP@0.50).

**Table 1:** Recent DL-based models and datasets for plant Disease detection.

Author (year)	Model	Dataset	No. of Images	No. of Classes	mAP@0.50 (%)
Tao et al. (2025) [13]	CEFW-YOLO (Improved YOLOv11)	Self-acquired apple leaf disease detection (ALD) dataset.	2452	4	87.3%
Sun et al. (2025) [3]	E-TomatoDet (Improved YOLOv8)	Self-acquired tomato leaf disease dataset.	4510	5	97.2%
Abulizi et al. (2025) [14]	DM-YOLO (Improved YOLOv9)	Tomato leaf dataset from Roboflow (Bryan, 2023)	4124	9	86.4%
Miao et al. (2025) [15]	SerpensGate-YOLOv8 (Improved YOLOv8)	PlantDoc dataset.	2598	27	64.9%
Ji *et al.* (2024) [16]	Improved YOLOv8	Subset of Plant Village dataset for tomato leaf disease.	10041	9	94.9%
Chen at el. (2024) [4]	YOLOv8-ACCW (Improved YOLOv8)	Self-collected grape leaf disease detection dataset.	10000	4	92.8%
Li et al. (2024) [18]	YOLO-Leaf (Improved YOLOv8)	Plant Pathology 2020-FGVC7 and Plant Pathology 2021-FGVC8 datasets	FGVC7: 1821 FGVC8: 18632	FGVC7: 4 FGVC8: 6	FGVC7: 93.88% FGVC8: 95.69%
Wang et al. (2024) [21]	Improved YOLOv6	Subset of Tomato leaf dataset from Roboflow (Bryan, 2023).	4659	6	97.2%
Umar et al. (2024) [22]	Improved YOLOv7	Self-collected tomato leaf disease dataset.	8337	7	98.8%
Wang et al. (2024) [5]	TDGA (Improved YOLOv5)	Tomato leaf disease detection dataset available at Kaggle.	4578	4	91.4%
Zhong et al. (2024) [23]	Improved YOLOv8	Hybrid tomato leaf disease dataset.	3362	9	85.6%
Meng et al. (2024) [24]	YOLOV5-CBAM-C3TR (Improved YOLOv5)	Publicly available apple leaf pathology image dataset.	3900	3	73.4%
Wang and Liu (2024) [26]	TomatoDet (Improved YOLOv8)	Self-collected dataset from a tomato cultivation facility in Shouguang City, Shandong Province, China.	2000	5	92.3%
Abid et al. (2024) [27]	YOLOv8n	Hybrid dataset of five major crops.	2850	19	98.3%
Luo at el. (2023) [28]	Light-SA YOLOV8 (Improved YOLOv8)	Self-collected Citrus diseases and Pests Dataset.	3202	6	92.6%
Yang et al. (2023) [29]	Improved YOLOv8s	Self-collected tomato leaf disease dataset.	3098	3	93.4%
Liu et al. (2023) [30]	Improved YOLOv5s	Self-acquired tomato brown rot disease dataset.	7900	2	94.6%
Zhang et al. (2023) [31]	M−AORANet	Hybrid tomato leaf disease dataset.	3123	6	96.47%
Chen at el. (2023) [32]	YOLO-GIC-C (Improved YOLOv5)	Hybrid strawberry disease detection dataset.	2246	7	94.7%
Zhu et al. (2023) [33]	EADD-YOLO (Improved YOLOv5)	Self-collected apple leaf disease dataset.	26377	5	95.5%

Despite significant progress in DL based plant disease detection, several gaps still exist. Existing literature works are based on self-collected datasets or subsets of public datasets. While some datasets are class-balanced [5,26,27,30], the majority exhibit class imbalance [14] – [16,21,22]. The rest of the research does not specify whether their datasets are class-balanced or not. This research aims to solve this issue. Our focus is to develop a hybrid dataset where classes are balanced. This will help our proposed model perform well and provide an unbiased performance for model evaluation. Another goal of this research is the trade-off between model efficiency and accuracy. While many improved YOLO models achieve high accuracy, some of them increase model complexity, making them less suitable for real-world deployment on resource-constrained devices [14,24,26,29,31]. This research work aims to improve detection accuracy and at the same time maintain or improve efficiency in terms of parameters, GFLOPS, and model size. In addition, the black box nature of DL models can present a barrier to their adoption in practical agricultural settings, where interpretability and trust are most important for users such as farmers and agronomists. Most of the agricultural-based DL models lack explainability [5,14] – [16,21,22,26,27,30]. This study integrates XAI techniques to provide overall clarity for making the model’s decisions transparent. Finally, given the economic importance of tomatoes as a global crop, developing a dedicated and highly accurate detection system for tomato leaf disease is highly useful for early prevention.

## 3. Materials and methods

This study introduces LeafDet, which is built upon a popular and powerful deep learning framework known as YOLOv8. Generally, YOLO models are well known for their ability to detect objects in images quickly. LeafDet is specifically designed to be highly effective at finding and identifying various tomato leaf diseases. To fix the class imbalance issue of tomato plant leaf disease, a hybrid dataset, PlantTom, is developed where images are collected from publicly available datasets. This dataset is balanced such that each disease type has a similar number of images. Since the hybrid dataset is prepared from multiple diverse sources, it will enhance the performance of DL models by exposing them to a wide range of data, thereby improving their generalizability. It will be shown later in this paper that the proposed model achieves high detection accuracy while keeping the number of parameters, GFLOPS, and model size low. This is crucial for real-time applications like mobile apps, drones or robots.

In this section, the dataset and the model development process with necessary performance metrics are described. In Section 3.1, the PlantTom dataset development procedure is given. Section 3.2 shows the performance metrics that are used in this study. Section 3.3 shows the details of the LeafDet model architecture with the descriptions of the modules that are used. Section 3.4 mentioned PIoUv2 loss function and Section 3.5 describes the methods used to train and test the model step by step. Finally, Section 3.6 describes model explainability via Eigen-CAM technique.

### 3.1 Development of PlantTom dataset

Class imbalance is a common issue in public datasets which can make it difficult to test models accurately. To fix this and ensure the proposed LeafDet model is tested properly, the PlantTom dataset has been developed. This dataset puts together images from different public datasets to ensure the classes are more balanced. The PlantTom dataset was created by combining images from popular public datasets like Plantdoc [34,35], Plantvillage [36,37], Tomato leaf disease dataset by Bryan [14,38], Fieldplant [39,40], and additional images collected from the Internet. The dataset consists of 8 classes, each representing tomato leaf disease: (i) Early Blight, (ii) Late Blight, (iii) Leaf Mold, (iv) Mosaic Virus, (v) Bacterial Spot, (vi) Septoria, (vii) Spider Mites and (viii) Yellow Leaf Curl Virus. Initially, this dataset consisted of 4819 images with 5805 instances. The dataset was partitioned into train, valid and test subsets where 3017 images were used for training, 1423 images for valid and 379 for test data (Table 2).

**Table 2:** PlantTom dataset summary.

Dataset split	No. of images (Before Augmentation)	No. of images (After Augmentation)
Training	3017	6034
Valid	1423	1423
Test	379	379
Total	4819	7836

The images are applied auto orientation techniques and then stretched to a resolution of 640 by 640 pixel as a preprocessing task. Then data augmentation techniques are applied to test the dataset in a variety of lighting and weather conditions and applied only to the training set to avoid data leakage. Horizontal and vertical flipping, brightness adjustment (±10%), and noise addition (up to 0.14% of pixels) augmentations are applied in this dataset. The development and augmentation of this PlantTom dataset have been done by using Roboflow platform [41].

**Fig 1:**
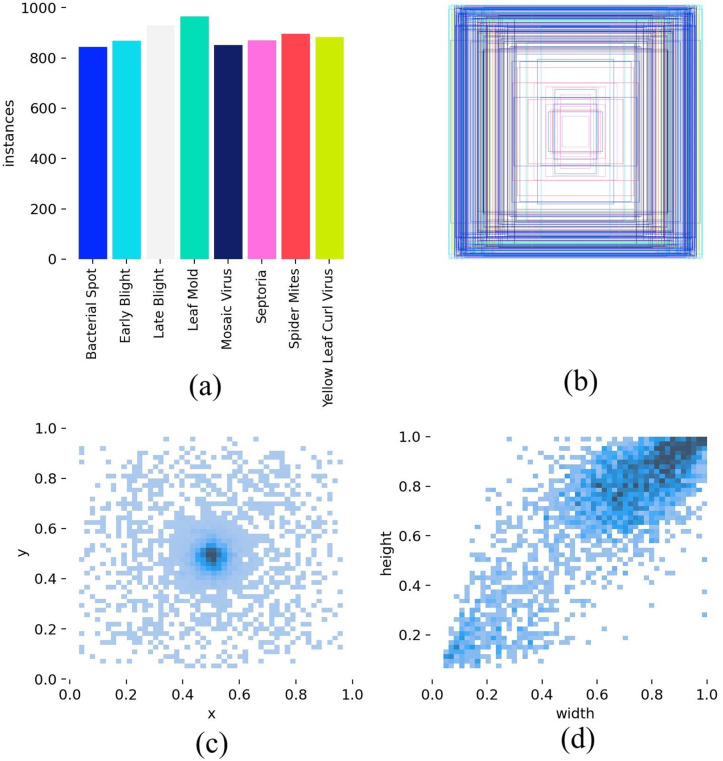
Analysis of PlantTom dataset labels. **(a)** Class distribution; **(b)** Bounding box distribution; **(c)** Heatmap of bounding box centers; **(d)** Heatmap of bounding box dimensions.

After applying these augmentations into training subset, the dataset extends to 7836 images with 9352 instances. In the augmented dataset, 77% (6034 images) allocated for training, 18% (1423 images) for validation, and the remaining 5% (379 images) for testing. The summary of the dataset and class-wise instance distribution in PlantTom dataset are given in Table 2 and Table 3 respectively. The analysis of this dataset like class wise and bounding box distributions, heatmap of bounding box centers and dimensions are shown in Fig 1.

**Table 3:** Class-wise instance distribution in PlantTom dataset.

Disease name	No. of instances (Before augmentation)				No. of instances (After augmentation)			
	Training	Valid	Test	Total	Training	Valid	Test	Total
Early Blight	433	198	104	735	866	198	104	1168
Late Blight	464	198	60	722	928	198	60	1186
Leaf Mold	482	188	65	735	964	188	65	1217
Mosaic Virus	425	262	43	730	850	262	43	1155
Bacterial Spot	421	248	52	721	842	248	52	1142
Septoria	434	216	68	728	868	216	68	1152
Spider Mites	447	212	57	716	894	212	57	1163
Yellow Leaf Curl Virus	441	230	57	728	882	230	57	1169

### 3.2 Performance metrics

The performance of object detection models is primarily evaluated using a set of metrics. These metrics are very important for figuring out how effectively the model works in real settings. In this study, standard performance metrics were used to evaluate the LeafDet model. It is based on the Pascal VOC evaluation method. Key metrics include Precision, Recall, F1 Score, and mean Average Precision (mAP@0.5). Intersection over Union (IoU) was used to decide whether a detection is correct, with 0.5 as the threshold like in Pascal VOC. The key performance metrics of the proposed model are as follows: [42]


$$\begin{array}{c} Precision=\frac{TP}{TP+FP} \end{array}$$ (1)

$$\begin{array}{c} Recall=\frac{TP}{TP+FN} \end{array}$$ (2)

$$\begin{array}{c} F\text{1}Score=\text{2}\times \frac{Precision\times Recall}{Precision+Recall} \end{array}$$ (3)

$$\begin{array}{c} AP=\int_{\text{0}}^{\text{1}}P(R)dR \end{array}$$ (4)

$$\begin{array}{c} mAP=\frac{\text{1}}{N}\sum_{i=\text{1}}^{N}{AP}_{i} \end{array}$$ (5)

$$\begin{array}{c} FPS=\frac{\text{1}\text{0}\text{0}\text{0}}{InferenceTime(inmillisecond)} \end{array}$$ (6)

where TP, TN, FP, and FN mean true positives, true negatives, false positives, and false negatives, respectively. Precision measures the accuracy of positive predictions. It is the ratio between True Positives (TP) and total predicted positives (TP + FP). Recall measures the model’s ability to find all relevant positive instances. It determines, out of all the actual objects present in the images, how many objects are successfully detected. The F1 score is a performance metric used to balance precision and recall. Average Precision (AP) represents a Precision-Recall (PR) curve in a single numerical value. AP is the integral of the precision-recall curve. Mean Average Precision (mAP) is the mean of the average precision scores across all classes. While Average Precision (AP) evaluates a model’s performance for a single class, mAP evaluates object detection models across multiple classes. The term mAP@0.5 refers to the mean average precision calculated at IoU threshold 0.5. It represents that a prediction is correct if the detected area covers at least 50% of the actual object. Moreover, the term mAP@0.5:0.95 (or mAP50-95) is the average of mAP across multiple IoU thresholds, ranging from 0.5 to 0.95 with a step of 0.05. Inference Time is the time taken to process a single image or frame and produce a prediction. Frames Per Second (FPS) is the number of images or frames the model can process per second. In addition, model parameters are vital for the evaluation of the performance of the model. The number of floating-point arithmetic operations required for a model during inference to process a single input is referred to the Floating Point Operations Per Second (FLOPs). The higher value of this metric indicates that the model requires more computations to make a single prediction.

### 3.3 LeafDet model architecture

In this study, the LeafDet model is proposed, which is built upon the concept of the YOLOv8 object detection framework developed by Ultralytics [7]. It is explicitly designed to detect diseases on plant leaves. The model includes several improvements to its structure, especially in the backbone and neck parts, to make the feature extraction and fusion process more robust. These changes help the model detect leaf diseases more effectively.

**Fig 2:**
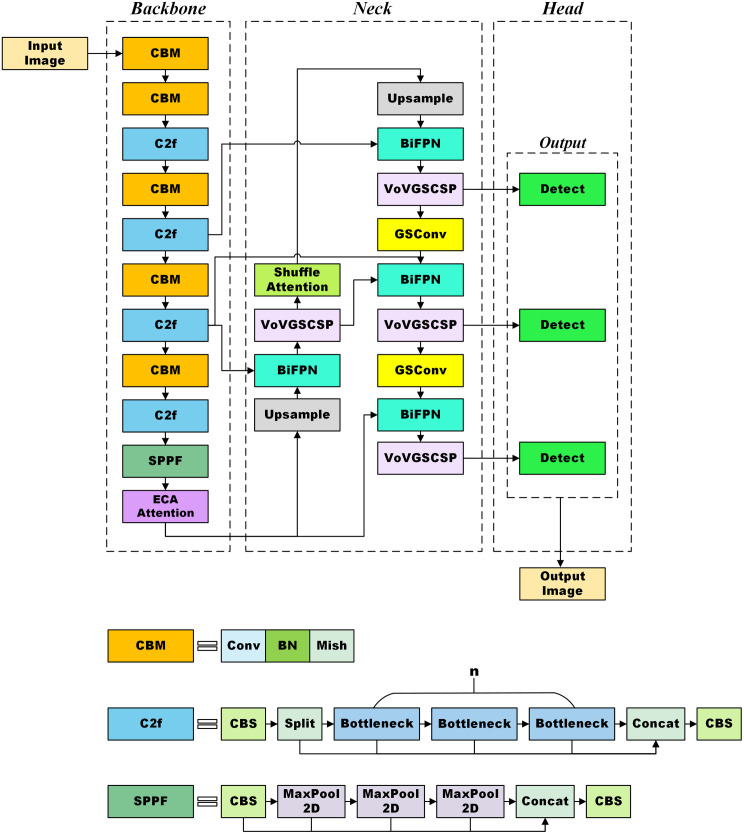
LeafDet model architecture.

The proposed LeafDet model architecture is shown in Fig 2. From the figure, it can be seen that several modifications have been made to the YOLOv8 model. In the backbone, these changes include CBM blocks with the Mish activation function, C2f modules, SPPF, and ECA Attention. In the neck, the modifications include BiFPN, VoVGSCSP with GS-Bottleneck and GSConv, and Shuffle Attention modules.

The backbone architecture improves YOLOv8 model by removing the Conv block and incorporating the CBM Block, which is a convolutional block comprising a Convolution layer, Batch Normalization, and Mish activation function [6]. Note that the Mish activation function is a self-regularized, non-monotonic function defined as:


$$\begin{array}{c} \begin{array}{c} f(x)=x*\text{tan }\text{h}\left(softplus(x)\right) \end{array} \end{array}$$ (7)

where, $softplus(x)=ln\left(\text{1}+e^{x}\right)$.

Next, C2f module [7] is integrated which enables efficient feature reuse through depth-wise separable convolution; SPPF (Spatial Pyramid Pooling Fast) module [7] is also added into the backbone, which aggregates multi-scale features at the end of the backbone. After the SPPF module, an ECA Attention (Efficient Channel Attention) module is embedded to strengthen channel-wise feature emphasis without dimensionality reduction [8]. It helps a neural network focus on important features in an image by using simple channel-wise weights. The attention weights in ECA attention are computed as:


$$\begin{array}{c} \begin{array}{c} Attention(c)=Conv\text{1}D\left(GAP\left(feature_{m}ap\right)\right) \end{array} \end{array}$$ (8)

where GAP = global average pooling and Conv1D is a small filter. Equation (8) describes how ECA Attention computes channel-wise attention weights by applying a 1D convolution to globally pooled features.

**Fig 3:**
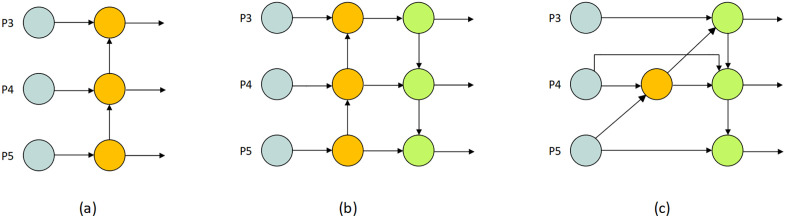
Evolution of feature pyramid networks: (a) FPN; (b) PANet; (c) BiFPN.

The neck architecture is designed for enhanced multi-scale feature fusion. In the neck part of the proposed model, BiFPN (Bidirectional Feature Pyramid Network) is used for top-down and bottom-up feature fusion with learned weights [9]. In LeafDet model, BiFPN is used to improve multi-scale features. It applies in LeafDet to avoid the vanishing gradients problem [43]. The skip connection technique is used to let BiFPN combine both old and new features, so no important detail is lost. Fig 3 provides a visual representation of Feature Pyramid Networks (FPN), Path Aggregation Networks (PANet), and Bidirectional Feature Pyramid Network (BiFPN).

In addition, the traditional C2f blocks in the neck are replaced with the VoVGSCSP module. This VoVGSCSP is a lightweight module that combines GSConv with a CSP (Cross Stage Partial) [10], replacing traditional C2f blocks for computational efficiency and deeper receptive fields, also reducing the number of parameters. Moreover, GSConv (Ghost Shuffle Convolution) module is used for improved channel-wise efficiency [10]; it also reduces the number of parameters. Shuffle Attention, positioned mid-neck, introduces both spatial and channel attention simultaneously for better context modeling [11].

**Fig 4:**
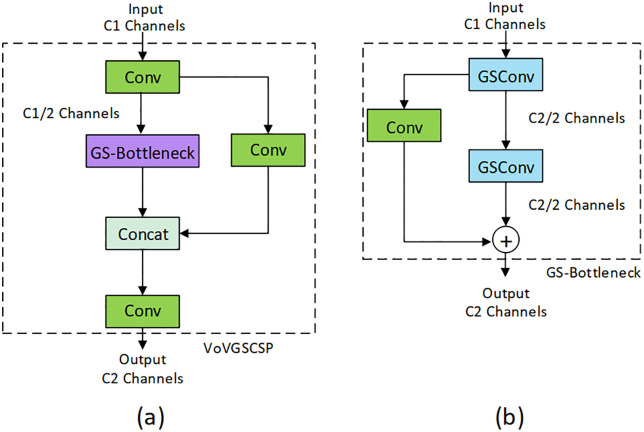
VoVGSCSP architecture: (a) VoVGSCSP; (b) GS-Bottleneck.

Another important change in the neck is the use of the shuffle attention module that combines both channel and spatial attention by splitting features into groups and mixing the outputs using a channel shuffle. For each group, the equation for the output feature y is denoted as: [44]


$$\begin{array}{c} y=[F_{\text{1}}(x)⊗x]\text{ ©}[F_{\text{2}}(x)⊗x] \end{array}$$ (9)

where ⊗ denotes element-wise product or tensor product, © denotes concatenation; F_1_ and F_2_ represent channel and spatial attention, respectively. Here feature mapping for each group’s input can be represented as $x\in R^{C\times H\times W}$. In this case, GSConv combines standard convolution and depthwise convolution, then shuffles the output channels [10]. This GSConv module is used in GS-Bottleneck, which is a part of the VoVGSCSP module. VoVGSCSP stands for VoVNet-based Ghost Shuffle Cross Stage Partial [10]. The architecture of this module is given in Fig 4.

Finally, the detection head architecture follows YOLOv8’s anchor-free detection model, which makes predictions at three different scales that match low, medium, and high-resolution features.

### 3.4 PIoUv2 loss function

The Intersection over Union (IoU) loss measures how much the predicted box overlaps with the actual annotated box. A higher IoU means better localization of the object.

In this research, PIoUv2 is used for LeafDet model to reduce the number of overlapped objects which improves the model’s detection ability. It extends the previously developed PIoU by adding an attention mechanism. The PIoU loss function is expressed as: [45]


$$\begin{array}{c} PIoU=IoU-\frac{\rho^{\text{2}}\left(c,c_{gt}\right)}{w^{\text{2}}+h^{\text{2}}}-\alpha .\frac{{\left(w_{gt}-w\right)}^{\text{2}}+{\left(h_{gt}-h\right)}^{\text{2}}}{\overset{\text{2}}{\underset{gt}{w}}-\overset{\text{2}}{\underset{gt}{h}}} \end{array}$$ (10)

where, c= center points of predicted box,

$c_{gt}=$ center points of the ground truth bounding box,

$\rho^{\text{2}}\left(c,c_{gt}\right)=$ Euclidean distance between predicted and ground truth box centers,

w= width of predicted box,

h= height of predicted box,

$w_{gt}=$ width of ground truth box,

$h_{gt}=$ height of ground truth box.

Equation (10) illustrates how the model enhances object detection by reducing overlap and more accurately matching the size and position of predicted boxes to the ground truth objects. The term PIoUv2 extends Eq. 10. by adding a non-monotonic attention technique. The equation of PIoUv2 is given as: [45]


$$\begin{array}{c} PIoUv\text{2}=\text{1}-PIoU+\lambda .\phi (IoU).\psi \left(\Delta_{geometric}\right) \end{array}$$ (11)

where, ϕ(IoU)= non-monotonic attention,

$\Delta_{geometric}=$ geometric inequality between predicted and ground truth boxes,

$\psi \left(\Delta_{geometric}\right)=$ geometric relationship penalties.

Equation (11) means that the term PIoUv2 improves localization by penalizing geometric misalignment and emphasizing meaningful overlaps.

### 3.5 Training and evaluation setup

The experimental setup for this study was configured on a system equipped with an Intel® Xeon® @2.20 GHz CPU, an NVIDIA Tesla V4 GPU, and 16 GB of RAM, running on the Ubuntu 20.04 LTS operating system. Pytorch 2.4.1 and CUDA 12.1 are used in this environment. The specific details of the environment setup are provided in Table 4.

**Table 4:** Environment setup for experiment.

Item	Description
CPU	Intel® Xeon® @2.20 GHz
GPU	NVIDIA Tesla V4 GPU
RAM	16 GB
OS	Ubuntu 20.04 LTS
Python Version	Python 3.10.12
Deep Learning Framework	Pytorch 2.4.1
Parallel Computing Architecture	CUDA 12.1

The LeafDet model was trained for 100 epochs and the batch size was selected to 16. The input image size was selected to 640 by 640 pixels. Stochastic Gradient Descent (SGD) was used as the optimizer, with an initial learning rate of 0.01, and a final learning rate was also set to 0.01. An early stopping patience was set to 100 epochs. Automatic Mixed Precision (AMP) was enabled during training to optimize memory usage and speed. Mosaic augmentation was disabled for the final 10 epochs, to enable stable convergence. Table 5 provides a detailed overview of the training and validation parameters.

**Table 5:** Training and validation parameters settings.

Parameter	Magnitude	Description
epochs	100	Number of training epochs
patience	100	Early stopping patience
batch	16	Batch size for training
imgsz	640	Input image size (height and width)
optimizer	SGD	Stochastic Gradient Descent (SGD) optimizer is used
device	cuda	Device to use: cpu, cuda, cuda:0
workers	8	Number of dataloader workers
close_mosaic	10	Disable mosaic augmentation for final 10 epochs
amp	True	Automatic Mixed Precision (AMP) during training
iou	0.6	Intersection over Union (IoU) threshold for NMS
max_det	300	Maximum number of detections per image

Default hyperparameter settings from Ultralytics were used in this study [7]. To ensure reproducibility during model training, a fixed random seed of 0 was used in all experiments.

### 3.6 Model explainability via Eigen-CAM

Eigen-CAM is a technique used to visualize the most significant part of an input image during inference via DL model. It works by analyzing the principal components (eigenvectors) of the gradient-weighted feature maps at a layer, highlighting the most important regions without needing class labels. Let, I denote the input image of the PlantTom dataset where image size $(i\times j)I\in R^{i.j}$ and $W_{L=n}$ denote the combined weight matrix of the first k layers of size (m,n). Then the class activated output for the last layer L=k of the DL model is given by: [12]


$$\begin{array}{c} O_{L=k}=\overset{T}{\underset{L=k}{W}}I \end{array}$$ (12)

Factorizing Eq. 12 that is, decomposing the output using singular value decomposition theory, we get,


$$\begin{array}{c} \overset{M\times N}{\underset{L=k}{O}}=U^{M\times M}\;\Sigma^{M\times N}\;{\left(V^{N\times N}\right)}^{T} \end{array}$$ (13)

where U is an M×M orthogonal matrix and Σ is an M×N diagonal matrix and V is an N×N orthogonal matrix. Here, the columns of U and V are the left singular vectors. Then, the class activation map is given by: [12]


$$\begin{array}{c} L_{Eigen-CAM}=O_{L=k}V_{\text{1}} \end{array}$$ (14)

where $V_{\text{1}}$ is the first eigen vector of V matrix. Equation (14) indicates that the Eigen-CAM heatmap is generated by projecting the output of the last layer of the DL model onto its most dominant feature direction.

## 4. Results and discussion

In this section, the outputs of LeafDet model and its transparency via XAI are discussed. In Section 4.1, the detailed experimental results for the proposed LeafDet model are given. Next, in Section 4.2, the output of Eigen-CAM is discussed.

### 4.1 Experimental results of LeafDet

This section discusses the findings of the proposed LeafDet model and its comparison with various other SOTA object detection models. The impact of different modules in the proposed model is analysed through an ablation study, and the performance of LeafDet with various IoU loss functions is evaluated. The class wise performance comparison is presented in Table 6. The proposed LeafDet model performs well on the PlantTom dataset and achieves an F1-score of 87.4% and mAP@0.5 of 91.6% across all classes. LeafDet model achieves an F1-score of 92.4% for ‘Yellow Leaf Curl Virus’ and 90.5% for ‘Leaf Mold’. Precision remains consistent across all classes. Recall varies slightly; in particular, ‘Early Blight’ disease and ‘Bacterial Spot’ disease led to lower F1-scores. Despite these variations, the model maintains overall better accuracy and low inference time, as well as provides a good score in other performance metrics.

**Table 6:** LeafDet model performance by class.

Class	P (%)	R (%)	F1 (%)	mAP@0.5 (%)
All	**93.7**	**81.9**	**87.4**	**91.6**
Bacterial Spot	96.3	77.8	86.1	88.9
Early Blight	87.2	75.3	80.8	86.4
Late Blight	90.9	81.3	85.8	92.4
Leaf Mold	93.3	87.8	90.5	95.5
Mosaic Virus	96.2	78.6	86.5	87.3
Septoria	94.6	81.0	87.3	91.8
Spider Mites	93.4	85.8	89.4	93.2
Yellow Leaf Curl Virus	97.5	87.8	92.4	97.0

**Fig 5:**
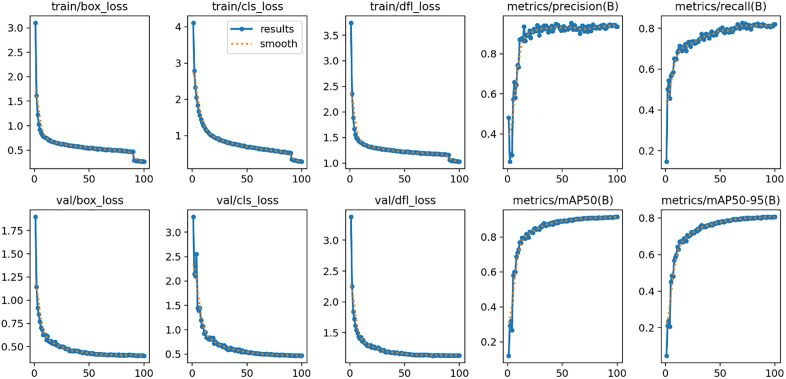
LeafDet model training results.

Fig 5 describes model performance during the training and validation steps. It shows box loss, class loss, and DFL loss during training and validation; it also indicates precision, recall, mAP50 and mAP50-95 for 100 epochs (values shown in Table 7).

**Table 7:** Summary of Training vs Validation metrics (values obtained from Fig 5).

epoch	train/box_loss	train/cls_loss	train/dfl_loss	metrics/precision(B)	metrics/recall(B)	metrics/mAP50(B)	metrics/mAP50-95(B)	val/box_loss	val/cls_loss	val/dfl_loss
1	3.102	4.111	3.743	0.481	0.147	0.121	0.047	1.897	3.315	3.379
10	0.757	1.287	1.401	0.734	0.648	0.728	0.598	0.628	1.071	1.428
20	0.649	0.953	1.316	0.915	0.726	0.831	0.707	0.517	0.720	1.260
30	0.605	0.846	1.277	0.932	0.736	0.860	0.743	0.477	0.637	1.219
40	0.576	0.761	1.252	0.940	0.752	0.881	0.763	0.455	0.587	1.183
50	0.537	0.683	1.222	0.926	0.798	0.889	0.778	0.429	0.538	1.161
60	0.525	0.652	1.212	0.921	0.798	0.900	0.789	0.415	0.506	1.137
70	0.502	0.604	1.190	0.914	0.827	0.908	0.796	0.416	0.494	1.140
80	0.487	0.558	1.181	0.934	0.814	0.910	0.801	0.409	0.477	1.134
90	0.468	0.519	1.165	0.929	0.817	0.911	0.805	0.410	0.478	1.129

**Fig 6:**
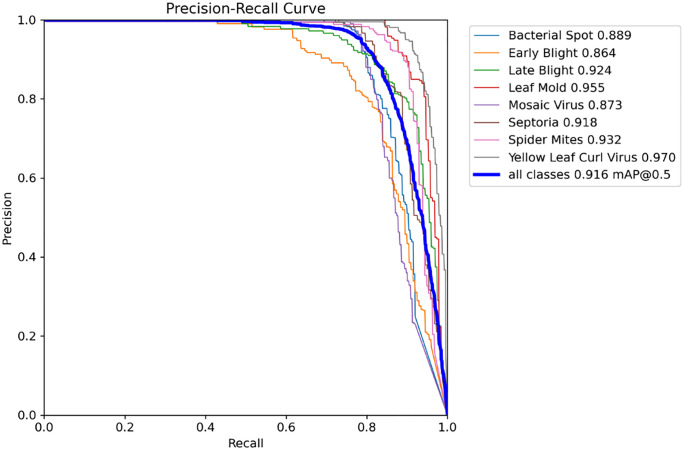
Precision-Recall curve.

Fig 6 shows the Precision-Recall Curve of the LeafDet model. The X-axis refers to recall, and the Y-axis refers to precision. This curve stays close to the top-right corner, indicating that the model is performing well. Table 8. summarizes the performance of LeafDet against several state-of-the-art object detection models like RetinaNet [46], Faster R-CNN [47], YOLOv3-tiny [48], YOLOv5n [49], YOLOv6n [50], YOLOv8n [51], YOLOv9t [52], YOLOv10n [53], YOLOv11n [54] and YOLOv12n [55]. These models were trained and evaluated on the same PlantTom dataset using identical preprocessing, splitting and evaluation protocol. Among the benchmarked models, LeafDet achieved the highest mAP@0.5 of 91.6%, surpassing the latest YOLOv12n (89.2%), YOLOv11n (90.5%) and YOLOv8n (89.4%). LeafDet achieves this accuracy with a low number of parameters (2.69 M) and GFLOPS (7.3G), and an inference time of 2.4 ms. In terms of model size, LeafDet is 5.44 MB, which is smaller than YOLOv8n (5.96 MB).

**Table 8:** Comparison of LeafDet (Proposed) models with SOTA models.

Model	Parameters (M)	GFLOPS	Inference time (ms)	Model Size (MB)	mAP@0.5 (%)
RetinaNet	37.69	93.64	80.8	277	86.0
Faster R-CNN	41.48	89.41	87.2	315	84.1
YOLOv3-tiny	12.13	18.9	2.5	23.20	85.5
YOLOv5n	2.50	7.1	2.2	5.03	89.2
YOLOv6n	4.23	11.8	1.8	8.29	88.0
YOLOv8n	3.01	8.1	2.1	5.96	89.4
YOLOv9t	1.97	7.6	3.0	4.43	89.9
YOLOv10n	2.70	8.2	3.1	5.49	89.8
YOLOv11n	2.58	6.3	2.3	5.22	90.5
YOLOv12n	2.56	6.3	3.8	5.27	89.2
LeafDet (Proposed)	**2.69**	**7.3**	**2.4**	**5.44**	**91.6**

Table 9 provides a direct comparison between the proposed LeafDet model and the YOLOv8n baseline model. LeafDet model achieves 91.6% mAP@0.5, which is a 2.2% improvement over the baseline YOLOv8n (89.4%). Additionally, LeafDet shows higher precision (P) at 93.7% versus 91.9%, recall (R) at 81.9% versus 79.7%, and F1-score at 87.4% versus 85.4%. Besides these, LeafDet has fewer parameters (2.69 M vs. 3.01 M), lower GFLOPS (7.3G vs. 8.1G), and a smaller model size (5.44 MB vs. 5.96 MB) while maintaining a comparable inference time (2.4 ms vs. 2.1 ms) compared with the baseline (YOLOv8n).

**Table 9:** Comparison of LeafDet model with YOLOv8n (Baseline).

Model	P(%)	R(%)	F1(%)	mAP@0.5 (%)	Parameters (M)	GFLOPS	Inference time (ms)	Model Size (MB)
YOLOv8n	91.9	79.7	85.4	89.4	3.01	8.1	2.1	5.96
LeafDet (Proposed)	**93.7**	**81.9**	**87.4**	**91.6**	**2.69**	**7.3**	**2.4**	**5.44**

To determine how each module impacted the overall performance of the LeafDet model, an ablation study was carried out with YOLOv8n as the base model. Table 10 details the results of this ablation study. The YOLOv8n model achieved a mAP@0.5 of 89.4%. To incorporate the PIoUv2 loss function alone improved the mAP@0.5 to 90.7%. Adding CBM, ECA Attention, Shuffle Attention, GSConv, VoVGSCSP, or BiFPN individually also showed improvements over the base model. Adding CBM resulted in a mAP@0.5 of 90.5%, while BiFPN achieved 90.9%. The combination of all proposed modules (PIoUv2, CBM, ECA Attention, Shuffle Attention, GSConv, VoVGSCSP, BiFPN) resulted in the highest mAP@0.5 of 91.6% (for a given split ratio as shown in Table 11). LeafDet also outperforms in terms of precision (93.7%) and F1-score (87.4%).

**Table 10:** Ablation study of the LeafDet model.

Base Model	PIoUv2	CBM	ECA Attn.	Shuffle Attn.	GSConv	VoVGSCSP	BiFPN	P (%)	R (%)	F1 (%)	mAP@0.5	Parameters (M)	GFLOPS	Inference time (ms)
YOLOv8n								91.9	79.7	85.4	89.4	3.01	8.1	2.1
		✔						93.1	80.4	86.3	90.5	3.01	8.1	2.3
			✔					93.1	81.3	86.8	90.7	3.01	8.1	2.1
				✔				93.4	80.0	86.2	90.3	3.01	8.1	2.0
					✔			93.2	81.3	86.8	90.9	2.92	8.0	2.1
						✔		91.9	81.1	86.2	90.2	2.77	7.3	2.0
							✔	93.5	81.6	87.1	90.9	3.02	8.1	2.2
	✔							92.7	82.2	87.1	90.7	3.01	8.1	2.1
	✔	✔						94.3	79.6	86.3	90.5	3.01	8.1	2.3
	✔		✔					93.2	82.6	87.6	90.9	3.01	8.1	2.2
	✔			✔				93.2	80.7	86.5	89.7	3.01	8.1	2.2
	✔				✔			94.3	80.7	87.0	90.9	2.92	8.0	2.2
	✔					✔		91.5	81.9	86.4	90.4	2.77	7.3	2.0
	✔						✔	91.8	82.3	86.8	90.9	3.02	8.1	2.3
	✔	✔	✔	✔	✔	✔	✔	**93.7**	**81.9**	**87.4**	**91.6**	**2.69**	**7.3**	**2.4**

**Table 11:** Performance comparison under different dataset split ratios.

Model	Split Ratio	Validation mAP@0.5	Test mAP@0.5
**LeafDet (Proposed)**	77:18:5	91.6	91.1
	70:20:10	91.7	92.2

The impact of different IoU loss functions on the performance of LeafDet is presented in Table 12. When PIoUv2 [45] is used as the IoU loss function, the proposed LeafDet model achieves its highest mAP@0.5 of 91.6%. Other IoU variants, such as CIoU [56], DIoU [57], EIoU [58], GIoU [59], SIoU [60], MDPIoU [61], and PIoU [62], are also evaluated. Even though these other IoU modules result in comparable outcomes (For instance, CIoU at 91.2% mAP@0.5), PIoUv2 achieves a good overall performance for LeafDet. For LeafDet, the parameters, GFLOPS, and inference time remained unchanged with any of the IoU modifications. It indicates that the accuracy (mAP@0.5) measures are influenced by IoU loss function.

**Table 12:** Performance of LeafDet model by using different IoU loss function.

Model	IoU	P(%)	R(%)	F1(%)	mAP@0.5(%)	Parameters (M)	GFLOPS	Inference time (ms)
LeafDet (Proposed)	CIoU	91.4	81.7	86.3	91.2	2.69	7.3	2.4
	DIoU	92.2	80.9	86.2	90.8	2.69	7.3	2.5
	EIoU	93.6	80.8	86.7	90.5	2.69	7.3	2.5
	GIoU	92.9	81.9	87.1	90.2	2.69	7.3	2.3
	SIoU	93.6	79.9	86.2	90.0	2.69	7.3	2.5
	MDPIoU	90.2	82.6	86.2	90.8	2.69	7.3	2.5
	PIoU	90.3	82.7	86.3	90.7	2.69	7.3	2.4
	PIoUv2	**93.7**	**81.9**	**87.4**	**91.6**	**2.69**	**7.3**	**2.4**

In the following we explain how the above-mentioned steps helped to overcome overfitting of the proposed LeafDet model. First, the PlantTom dataset was carefully balanced to minimize class imbalance, reducing bias and improving generalization. Second, training versus validation monitoring, as shown in Fig 5 and Table 7, demonstrates that losses and metrics converge smoothly without divergence, indicating stable learning. Third, we assessed generalization through an independent re-split of the dataset using a 70:20:10 ratio. In Table 11, the results show the performance remains consistent across both validation and unseen test sets, with mAP50 scores of 91.7% (validation) and 92.2% (test) for the 70:20:10 split. These results closely match the original 77:18:5 split, which achieves 91.6% on validation and 91.1% on test, confirming negligible differences. Finally, systematic improvements across modules and loss functions (Tables 10 and 12) indicate performance gains are architectural, not due to overfitting.

Mish activation function performs better on many existing works. D. Mishra [6] shows that Mish can outperform many SOTA models in terms of accuracy, FLOPS, Inference Time, etc. Mish activation function has smooth non-monotonicity, which improves gradient flow and generalization. Evaluation of LeafDet under varying activation functions shows constant parameters (2.69M) and model size (5.44 MB), with minor differences in complexity and speed. Mish achieves the highest accuracy (mAP@0.5: 91.6%) over ReLU and SiLU (90.7%), indicating a reliable accuracy (Table 13). Runtime memory usage was not separately measured in this study; therefore, activation functions were compared using parameters, GFLOPS, inference time, model file size, and mAP@0.5.

**Table 13:** Performance of LeafDet model using different activation functions.

Activation function	Parameters (M)	GFLOPS	Inference time (ms)	Model Size (MB)	mAP@0.5 (%)
ReLU	2.69	7.3	2.1	5.44	90.7
SiLU	2.69	7.2	2.3	5.44	90.7
Mish	2.69	7.3	2.4	5.44	91.6

### 4.2 Model explainability via XAI: Eigen-CAM

**Fig 7:**
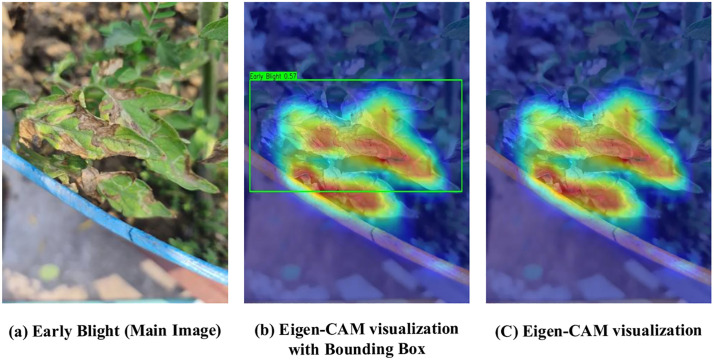
Model explainability by using Eigen-CAM. The original image in Fig 7(a) was captured by the authors for this study and is used with permission.

In this study, the Eigen-CAM method is used to interpret the decision of the LeafDet model [12]. The method works by analyzing the main features from the last convolutional layer of the model and projecting them back onto the image. To demonstrate model interpretability, Eigen-CAM is applied to a real-world Early Blight image captured by the authors using a Samsung Galaxy M62 smartphone from Chuadanga, Bangladesh (Fig 7). As shown in Fig 7(a), the original image contains visible symptoms such as lesion and discoloration. The model correctly localizes the infected region with a predicted class label and confidence score (Fig 7(b)). The corresponding Eigen-CAM visualizations in Fig 7(b)–(c) highlight the lesion-affected areas, showing strong alignment between the detected region and high-activation zones. This consistency indicates that the model focuses on disease-relevant features rather than background information.

## 5. Conclusion

In this research, an object detection model is developed based on the YOLOv8 architecture called LeafDet. This model is specifically designed for the reliable and efficient detection of tomato leaf diseases. An essential contribution of this study was the creation of the balanced PlantTom dataset. This dataset precisely addressed the class imbalance issues common in public datasets, thereby providing an unbiased foundation for model training and evaluation. The proposed LeafDet model achieves a mean Average Precision (mAP@0.5) of 91.6% on this custom-built dataset. This result is a 2.2% improvement over the baseline YOLOv8n model, which recorded 89.4% mAP@0.5. The model also outperforms the other state-of-the-art models, including the latest YOLOv11n and YOLOv12n, while maintaining a lightweight and efficient architecture with fewer parameters, lower GFLOPS, and a smaller model size. The ablation study clearly shows the positive impact of each integrated architectural enhancement, including CBM, C2f, SPPF, ECA Attention, BiFPN, VoVGSCSP, GSConv, and Shuffle Attention. Furthermore, the analysis of various IoU loss functions identified PIoUv2 as the optimal choice as a loss function for this specific application, contributing to the model’s peak performance. Furthermore, Eigen-CAM is used to visualize and validate the strength of LeafDet’s predictions. LeafDet offers a highly practical and deployable solution for automated disease detection in tomato cultivation. Its high accuracy and lower model size make it a more efficient and interpretable model. It can be a valuable tool for farmers and agricultural practitioners.

In future work, the model can be improved to handle multi-crop and multi-disease detection. This would make it more useful for a wide range of agricultural applications. Additionally, it can also be integrated with mobile devices or drones for real-time monitoring in the field. Such integration would help farmers identify diseases early and take quick action. Further research could explore continual learning methods. These would allow the model to adapt to changing disease patterns over time.
